# Delayed Presentation of Acute Compartment Syndrome After Isolated Closed Fibular Shaft Fracture: A Case Report

**DOI:** 10.7759/cureus.55850

**Published:** 2024-03-09

**Authors:** Dimitrios Giotis, Vasileios Panagiotopoulos, Sotiris Plakoutsis, Dimitrios Vardakas, Christos Konstantinidis

**Affiliations:** 1 Orthopaedic Department, General Hospital of Ioannina "G. Hatzikosta", Ioannina, GRC

**Keywords:** operative management, fasciotomies, isolated fibular fracture, delayed presentation, compartment syndrome

## Abstract

Post-traumatic compartment syndrome in the lower extremity has been commonly associated with fractures of the tibia. Only in rare cases, this critical condition might be related to isolated fibular fractures. We present a rare case of delayed onset of acute compartment syndrome after a solitary fracture of the fibula. A 40-year-old man with a history of coagulation disorders due to hepatic cirrhosis was admitted to a neighboring hospital after a car accident with left-sided fractures to ribs 9 and 10 and a transverse fracture in the mid-shaft of the left fibula. He was discharged from the hospital five days later with a posterior long leg splint and anticoagulant therapy. However, three days after discharge, he was seen in the emergency department of our hospital with severe pain and extensive swelling in the left leg. Weak posterior tibial and dorsalis pedis pulse in the right foot were detected. Moreover, sensory disturbances were found in the tibia and foot. Passive hallux dorsiflexion and plantar flexion were causing acute intense pain. A triplex ultrasound was negative for deep vein thrombosis. Apart from the clinical findings, the diagnosis of compartment syndrome was confirmed after evaluating intracompartment pressure measurements. The patient was taken emergently to the operating room for four-compartment fasciotomies. A large intramuscular hematoma was evacuated. Skin closure was accomplished in two stages within two weeks. Six weeks postoperatively, there was no sign of compartment syndrome sequelae and the patient was free of symptoms without any neurovascular deficiency in the operated limb and walked without crutches. Ten weeks later, he returned to his pre-injury daily activities. Although the majority of compartment syndrome cases are reported after high-energy trauma, patients with both coagulation disturbances and anticoagulation treatment are at higher risk of developing compartment syndrome secondary to simple fracture patterns.

## Introduction

Compartment syndrome is a clinical condition that occurs when the pressure within a closed fascial space elevates to high levels, resulting in a lack of local capillary perfusion to the soft tissues within the space. Persistence of this increased pressure due to bleeding or edema for a few hours might cause progressive tissue ischemia and consequently potential necrosis of the muscles and peripheral nerves, leading to even irreversible loss of limb function [1-4].

Regarding the tibia, compartment syndrome might be the result of numerous traumatic or nontraumatic etiologies. The most common cause is a tibial fracture, especially following a crush injury to the limb [1,2]. Younger age, high-energy trauma, and comminution of fracture have been reported as risk factors for the development of compartment syndrome after a fracture in the tibia [1,5,6]. However, this potentially devastating syndrome can be also associated with hemorrhagic disorders (coagulopathy, hemophilia, and liver cirrhosis), anticoagulant therapy, and burns, amongst others [1,2].

Prompt diagnosis and early treatment are crucial to avoid catastrophic consequences [7]. Frequently, the diagnosis is based on only clinical signs [7]. Initially, palpable tightness, swelling, and increased pain of the tibia on passive stretch followed by progressive paresthesia can be observed in clinical examination [8,9]. But there are cases where only a few signs are present [9]. If it is possible, the quantification of intramuscular pressure by direct continuous measurement would strengthen the validity of the diagnosis [10]. The operative management demands immediate decompressive surgical fasciotomies to decrease the intracompartment pressure.

The purpose of the current case report is to present an extremely rare case of delayed onset of acute tibial compartment syndrome after a solitary fibular shaft fracture in a patient with a history of coagulation disorders. We highlight the diagnostic process that was followed to recognize the delayed development of this clinical manifestation and the required operative management that was performed for a successful outcome.

## Case presentation

**Figure 1:**
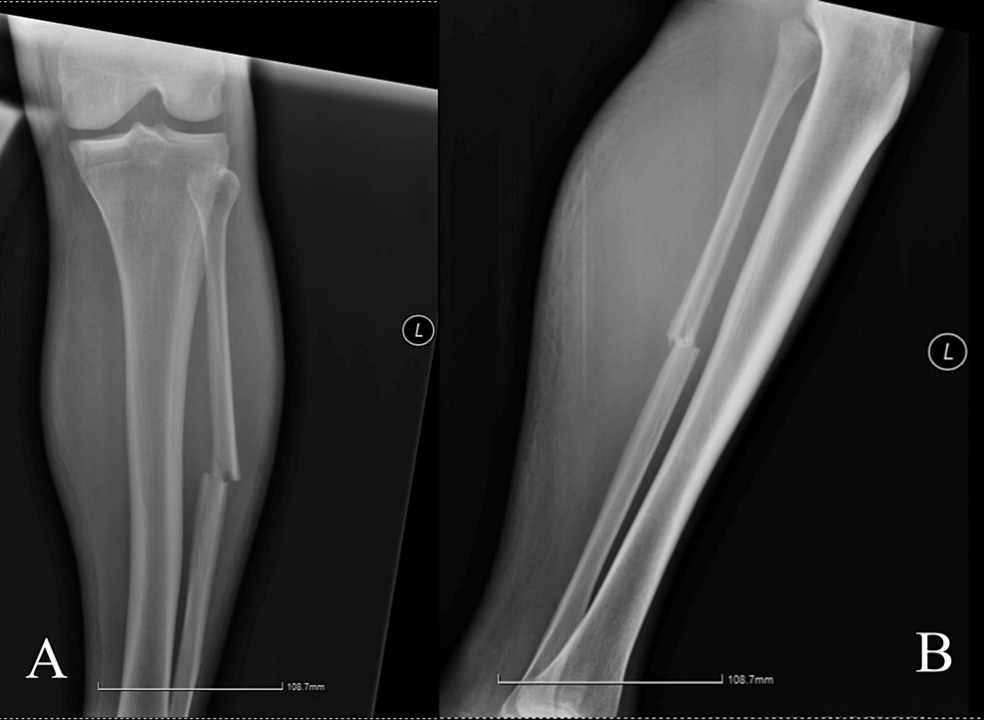
Left tibia radiograph showing anteroposterior (A) and lateral (B) views of the isolated fibular shaft fracture.

A 40-year-old male was admitted to another institution after sustaining a mid-shaft left fibula fracture due to a car accident. He had concomitant nondisplaced 9th and 10th left rib fractures. His medical history involved coagulation disorder as a result of liver cirrhosis (Figure 1). The patient was discharged five days later with a posterior long leg splint and anticoagulant treatment with low-molecular-weight heparin in a prophylactic dose.

**Figure 2:**
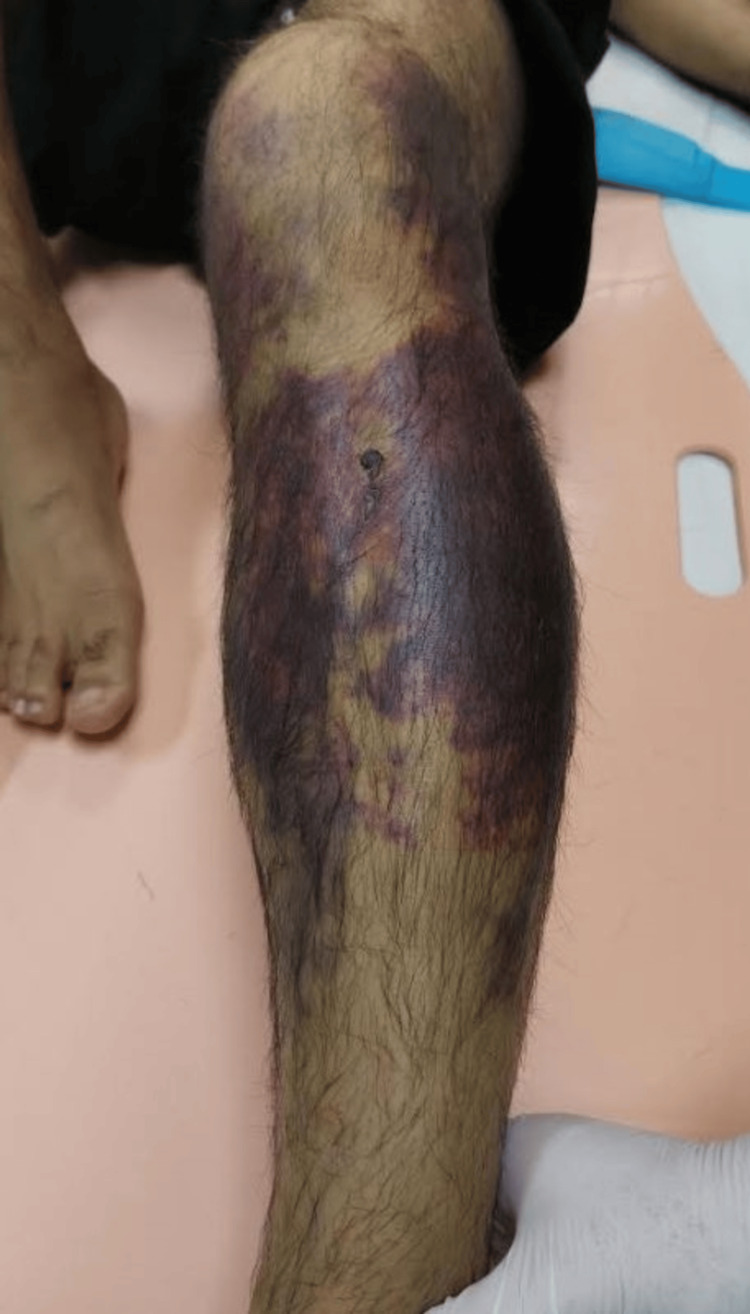
Discoloration of the skin with bruises, covering the majority of the anterior and posterior surface of the tibia.

However, three days later, he arrived at the emergency department of our hospital with severe pain and extensive swelling in the left calf. The pain was intense, especially on passive plantar flexion of his ankle. The muscles of all compartments were swollen, tense, and tender to palpation. The discoloration of the skin was obvious with extended seven- to 10-day-old bruises, covering the majority of the anterior and posterior upper surface of the tibia, indicating the presence of large hematomas (Figure 2). Neurovascularly, weak peripheral pulses were found in both the left posterior tibial and dorsalis pedis arteries. Moreover, sensory disturbances, such as numbness and decreased sensation to light touch over the anterior and posterior aspects of the tibia and dorsum of the foot, were observed. Regarding the motor strength assessment, the patient demonstrated grade 2 of 5 for the tibialis anterior, extensor, flexor hallucis longus, and gastrocnemius-soleus complex muscles.

The results of the laboratory tests conducted on admission are presented in Table 1. The patient displayed low hemoglobin levels and platelet count was less than 80,000. The international normalized ratio (INR) was slightly high, but prothrombin time (PT) and activated partial thromboplastin time (aPTT) were within normal levels. In parallel, the levels of liver enzymes, alanine aminotransferase (ALT), and aspartate aminotransferase (AST) were mildly elevated (Table 1).

**Table 1:** Laboratory test results.

Test	Value	Reference range	Units
Hemoglobin (Hgb)	9.5	13-17.5	g/dL
Platelets	78,000	150,000-400,000	Platelets per microliter (mcL)
International normalized ratio (INR)	1.37	0.8-1.2	International normalized ratio
Prothrombin time (PT)	12.5	10-13	Seconds
Activated partial thromboplastin time (aPTT)	35	25-36	Seconds
Creatinine kinase (CK)	1,308	40-320	IU/L
Alanine aminotransferase (ALT)	54	10-35	IU/L
Aspartate aminotransferase (AST)	51	10-35	IU/L
Albumin	3.4	3.4-5.4	g/dL
Total bilirubin	1.9	0.1-1	mg/dL
Urea	34	11-54	mg/dL
Creatinine	0.7	0.6-1.2	mg/dL

**Figure 3:**
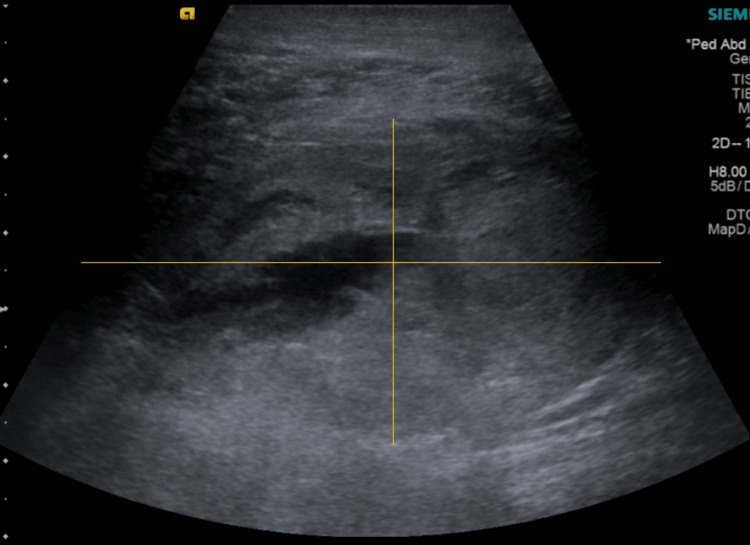
Ultrasound imaging displaying a large hematoma in the superficial posterior compartment.

The triplex ultrasonography was negative for deep vein thrombosis, but an additional evaluation revealed the presence of extensive hematomas mainly in the anterior and superficial posterior compartments (Figure 3). Additionally, intracompartment pressure measurements were performed in the affected limb. It was noted that the difference between the diastolic blood pressure and compartmental pressure (delta P) was less than 30 mm Hg in all the measured compartments, which strengthened our suspicion. Based on the findings mentioned above, along with the clinical and imaging findings, the diagnosis of compartment syndrome was made.

**Figure 4:**
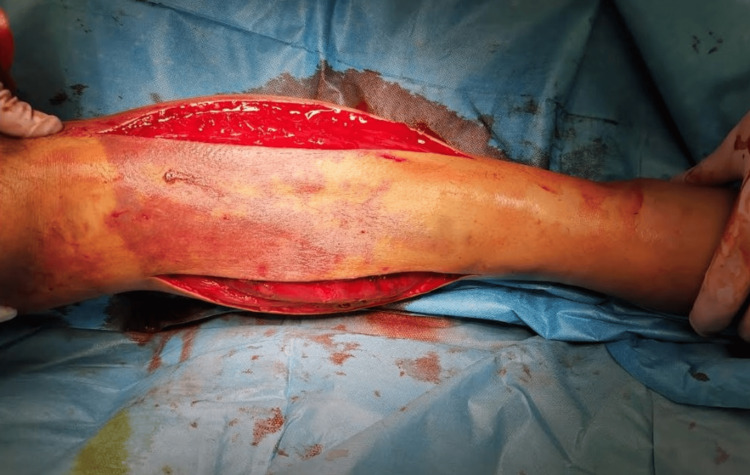
Two-incision four-compartment fasciotomy.

Therefore, the patient was taken to the theater, and emergent decompressive fasciotomies (anterolateral and posteromedial) to all four compartments of the left tibia were performed with the removal of a large intramuscular hematoma from the anterior compartment and a smaller one from the superficial posterior one (Figure 4). All the muscles, including those in the other two compartments (lateral and deep posterior), were under considerable tension but they were found viable, without demonstrating evidence of severe necrosis. Furthermore, the wounds were left open and compression dressing of the fasciotomy site was applied. No anticoagulant therapy was given. In parallel, regarding the antibiotic treatment, intravenous (IV) administration of amikacin and cefoxitin was started preoperatively.

On the first postoperative day, the patient received one unit of packed red blood cells (PRBCs) and one unit of fresh frozen plasma (FFP) due to low hemoglobin levels and intraoperative observation of increased bleeding. However, as a consequence of the inappropriate response of the hemoglobin levels and continuing low platelet count, repeated transfusions of two more units of PRBCs and four units of FFP coupled with vitamin K administration were required over the next two days to control bleeding and stabilize the hemoglobin levels.

**Figure 5:**
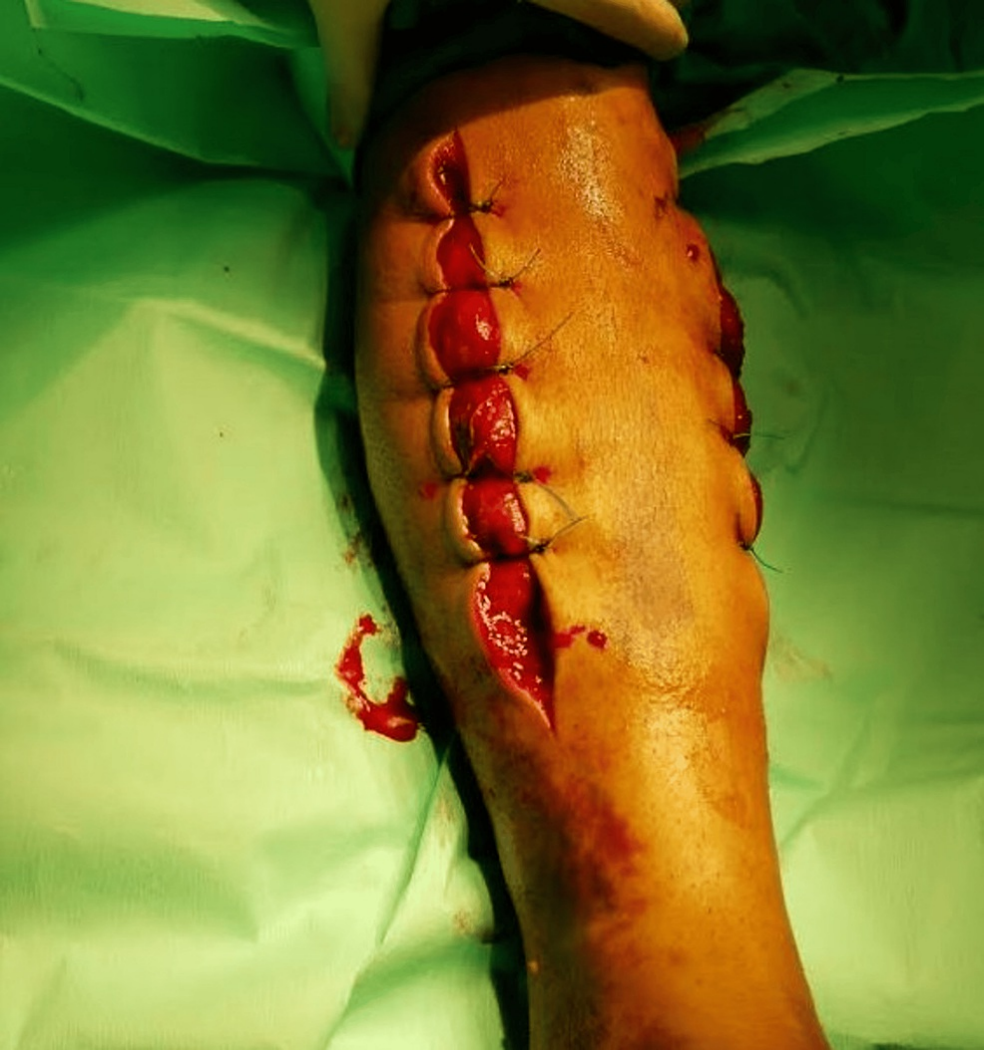
Primary closure of the fasciotomy wounds.

**Figure 6:**
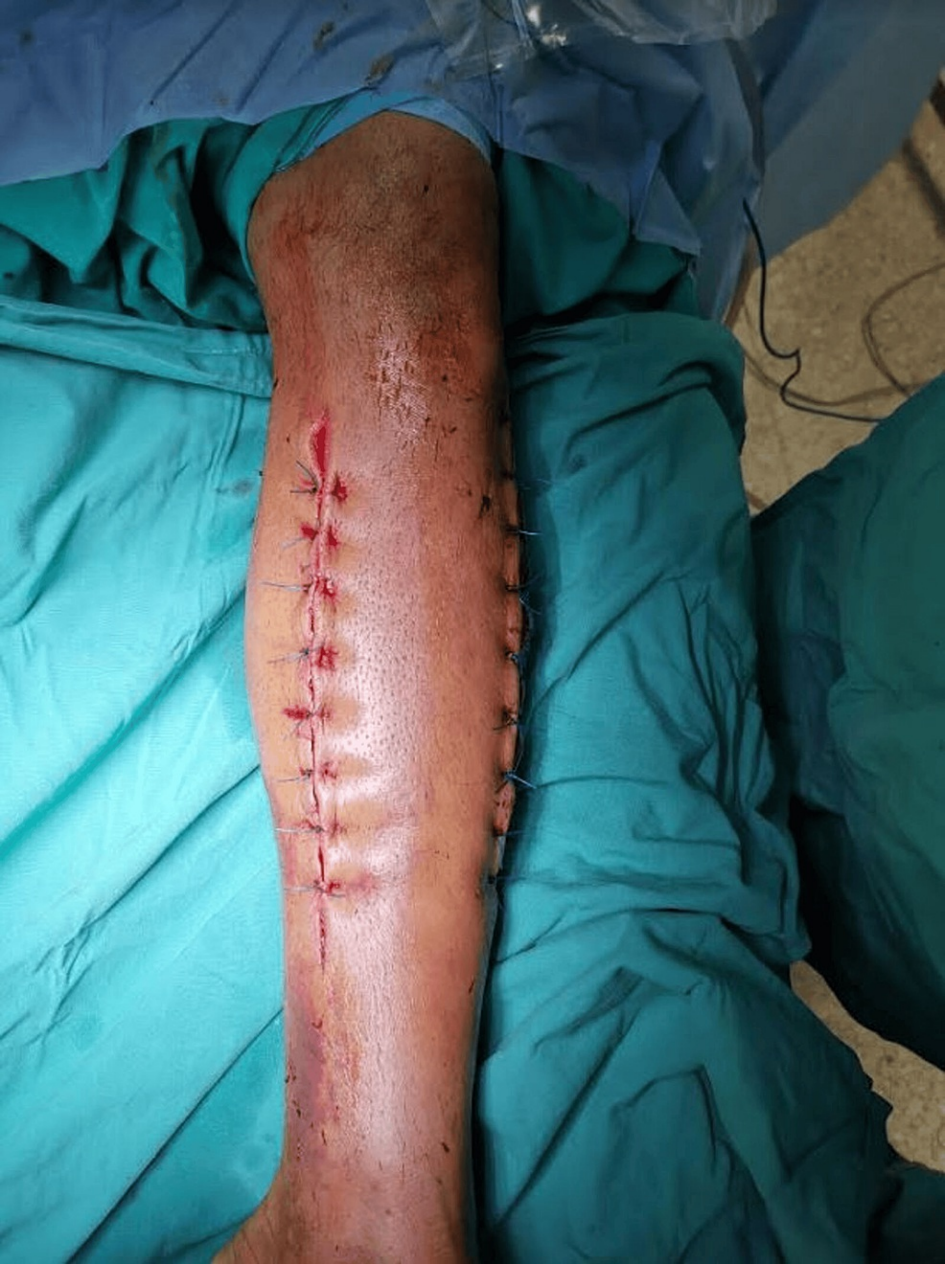
Second-stage skin closure.

Postoperatively, he had prompt relief of pain and remarkable improvement in skin color and motor and sensory function. On postoperative day three, the patient was taken back to the operating room for delayed primary closure of the wounds (Figure 5). Concurrently, debridement of necrotic skin and subcutaneous tissue was performed. Moreover, intraoperative cultures that had been obtained were negative, and IV antibiotics were discontinued on postoperative day five. The skin closure was completed in two stages within two weeks (Figure 6). Four weeks after the fasciotomies, the sutures were removed and the patient was discharged from the hospital. For the next three weeks, he was scheduled for close follow-up. Six weeks after the initial operation, the patient walked without crutches and he was free of symptoms without residual neurovascular deficits in the affected limb or signs of compartment syndrome sequelae. Ten weeks later, he presented full muscle strength equal to the contralateral extremity and managed to return to his pre-injury level of activities.

It should be noted that the patient was informed that data regarding his case were submitted for possible publication and he provided written consent.

## Discussion

Acute compartment syndrome is considered an orthopedic time-sensitive emergency with potentially devastating manifestations. Regarding the tibia, it is a complication that is mostly associated with tibial fractures [1,2,10,11]. To our knowledge, there is only one study in the literature that reports the development of compartment syndrome after an isolated fibula fracture. In that study, the authors demonstrated a rare case of acute compartment syndrome after an isolated closed transverse fibular shaft fracture in a patient receiving dual anticoagulation therapy, that was treated successfully with four-compartment fasciotomies [12].

We also present an incidence of acute compartment syndrome after an isolated fibula fracture. It is worth noting that the delayed onset of compartment syndrome, occurring over one week after the initial high-energy injury, is an extremely rare occurrence and warrants particular attention. It should be also underscored that the patient had a history of coagulation disorders due to liver cirrhosis. He had thrombocytopenia and was under low-molecular-weight heparin in prophylactic dose, which could be related to the onset of compartment syndrome. On the other hand, the tests of the coagulation cascade that were performed revealed nearly normal levels regarding the blood clotting function.

In general, compartment syndrome has been described in cirrhotic patients with coagulopathy, after a minor trauma or even spontaneously [13]. In fact, these patients demonstrate a higher risk of bleeding, especially during an operation or procedure as they frequently have a combination of coagulation defects due to impaired hepatic synthesis, such as decreased synthesis of coagulation or thrombocytopenia from hypersplenism, and thus, it is hard to control bleeding [13]. In cases of fasciotomies, repeated transfusion of FFP and other blood products might be needed to correct the coagulopathy and achieve hemostasis [13]. Uncontrollable bleeding has been reported after fasciotomies in cirrhotic patients with fatal results and surgeons should always be aware of such detrimental complications, particularly when these patients might receive anticoagulants [14].

Hopefully, in our case, there was no reduction in renal or liver function as depicted in consecutive laboratory blood tests, and postoperative bleeding was successfully controlled with repeated transfusions of PRBCs and FFP.

Concerning the diagnostic approach, there is no established single threshold level of intracompartment pressure agreed upon in the literature for accurate diagnosis of compartment syndrome, and therefore, the diagnosis is mostly clinical with or without intracompartment pressure monitoring [15-18]. In our patient, the evaluation of patient symptoms and clinical examination findings along with intracompartment pressure monitoring was the keystone for the detection of compartment syndrome with delayed presentation.

Furthermore, as reported in the literature, definitive treatment for the majority of compartment syndromes includes an immediate decompressive fasciotomy in an attempt to release the compressed soft tissues, decrease the intracompartment pressure, and restore perfusion, followed by secondary closure of the wound [10,15]. As it is considered that muscle necrosis may occur within the first two hours of injury and irreversible complications, within the first six hours of the onset of symptoms, it is widely regarded that early decompression might be critical to achieve the best result [2,7,8,15,16]. Similarly, in our patient, a four-compartment fasciotomy was conducted, accompanied by a two-stage skin closure within two weeks, with a remarkable outcome. However, special care was given to the management of the patient's coagulopathy to avoid uncontrollable bleeding.

Conclusively, through this report, we aim to highlight that clinicians should have a high level of suspicion for the onset of compartment syndrome in patients with known coagulopathy or administration of anticoagulation, even in cases of uncommon anatomic locations, unusual injury mechanisms, or lack of fractures [15,16,18].

## Conclusions

In the lower extremities, compartment syndrome is typically reported after high-energy trauma that involves tibial fractures. However, patients who are given anticoagulants or have coagulation disorders are at a higher risk of developing compartment syndrome after simple injury patterns such as an isolated fracture of the fibula, even with a delayed onset. Thus, all clinical doctors must have a high index of suspicion after similar types of injury. Accurate diagnosis, emergency decompressive fasciotomies, and successful postoperative management of coagulopathy or anticoagulant treatment can provide satisfactory results in similar cases.
